# A Novel Cold-adapted *Methylovulum* species, with a High C16:1ω5c Content, Isolated from an Arctic Thermal Spring in Spitsbergen

**DOI:** 10.1264/jsme2.ME20044

**Published:** 2020-06-12

**Authors:** Tajul Islam, Øivind Larsen, Nils-Kåre Birkeland

**Affiliations:** 1 Department of Biological Sciences, University of Bergen, Bergen, Norway; 2 Bergen Katedralskole, Kong Oscars gate 36, 5017 Bergen, Norway; 3 NORCE Norwegian Research Centre AS, Bergen, Norway

**Keywords:** arctic spring, cold adapted, fatty acids, methanotrophs, pMMO

## Abstract

A novel cold-adapted methane-oxidizing bacterium, termed TFB, was isolated from the thermoglacial Arctic karst spring, Trollosen, located in the South Spitsbergen National Park (Norway). The source water is cold and extremely low in phosphate and nitrate. The isolate belongs to the *Methylovulum* genus of gammaproteobacterial methanotrophs, with the closest phylogenetic affiliation with *Methylovulum miyakonense* and *Methylovulum psychrotolerans* (96.2 and 96.1% 16S rRNA gene sequence similarities, respectively). TFB is a strict aerobe that only grows in the presence of methane or methanol. It fixes atmospheric nitrogen and contains Type I intracellular membranes. The growth temperature range was 2–22°C, with an optimum at 13–18°C. The functional genes *pmoA*, *mxaF*, and *nifH* were identified by PCR, whereas *mmoX* and *cbbL* were not. C16:1ω5c was identified as the major fatty acid constituent, at an amount (>49%) not previously found in any methanotrophs, and is likely to play a major role in cold adaptation. Strain TFB may be regarded as a new psychrotolerant or psychrophilic species within the genus *Methylovulum*. The recovery of this cold-adapted bacterium from a neutral Arctic thermal spring increases our knowledge of the diversity and adaptation of extremophilic gammaproteobacterial methanotrophs in the candidate family “*Methylomonadaceae*”.

*Aerobic* methane oxidation is performed by a unique group of methylotrophic bacteria found in various methane-containing environments, *e.g.* soil, lake sediments, rice paddies, landfills, tundra, and thermal and cold ecosystems (Hanson and Hanson, 1996; Dunfield *et al.*, 2007), in which they play an important role in controlling the release of methane into the atmosphere (Conrad, 1996; Reeburgh, 2007). Most methane in nature is formed through anaerobic microbial processes by methanogenic archaea. However, thermogenic methane, a non-microbial source, is generated through the abiotic decomposition of buried organic materials and geochemical processes. This geological methane is freed to the atmosphere via seeps, mud volcanoes, gas venting, and geothermal areas (Etiope and Klusman, 2002; Fiebig *et al.*, 2007; Nazaries *et al.*, 2013).

Methane-oxidizing bacteria (MOB), or methanotrophs, possess highly specialized metabolism and have the ability to grow using only methane and a few other one-carbon compounds as their source of carbon and energy. These microorganisms currently belong to the phyla *Proteobacteria*, *Verrucomicrobia*, and *Methylomirabilaeota* (the candidate phylum NC10) (Houghton *et al.*, 2019). A reclassification of the phylum *Proteobacteria* has more recently been recommended, which includes twenty-seven genera of proteobacterial methanotrophs (five genera of the class *Alphaproteobacteria* and twenty-two genera of the class *Gammaproteobacteria*) (Rahalkar *et al.*, 2007; Trotsenko and Murrell, 2008; Deutzmann *et al.*, 2014; Hirayama *et al.*, 2014; Khalifa *et al.*, 2015; Tavormina *et al.*, 2015), and two genera of the phylum *Verrucomicrobia* were also suggested (Op Den Camp *et al.*, 2009; Sharp *et al.*, 2014; Van Teeseling *et al.*, 2014). The taxonomic structure at the family level, within the order *Methylococcales* of gammaproteobacterial methanotrophs, was reconstructed using genome-based phylogeny, and three family lineages were proposed as *Methylomonadaceae* (Type Ia) (Parks *et al.*, 2018), *Methylococcaceae* (Type Ib) (Orata *et al.*, 2018), and *Methylothermaceae* (Type Ic) (Hirayama *et al.*, 2014).

Several key functional genes, *pmoA* (encoding a subunit of the particulate methane monooxygenase: pMMO), *mmoX* (encoding a subunit of the soluble methane monooxygenase: sMMO), and *mxaF* (encoding the large subunit of methanol dehydrogenase: MDH), have been used to identify aerobic gammaproteobacterial methanotrophs from various environments. The *pmoA* gene is often used as a phylogenetic marker for the detection of aerobic methanotrophs in distinct habitats, including cold methane seeps in the floodplains of west Siberian rivers (Knief, 2015; Oshkin *et al.*, 2016). The alphaproteobacterial genera (Type IIb) *Methylocella* (Dedysh *et al.*, 2004) and *Methyloferula* (Vorobev *et al.*, 2011) were described as psychrotolerant and acidophilic methanotrophs. They were isolated from cold ecosystems (wetlands) and only contain sMMO enzyme systems.

Methanotrophs generally occur in relatively cold environments, *e.g.* tundra soil, ground waters, arctic wetland soil, saline meromictic lakes, polar lakes, permafrost, and marine sediments (Bowman *et al.*, 1997; Trotsenko and Khmelenina, 2005; Wartiainen *et al.*, 2006). Until now, only two true psychrophilic species (optimal growth temperatures between 3.5 and 13°C), *Methylosphaera hansonii* (Bowman *et al.*, 1997) and *Methylobacter psychrophilus* (Omelchenko *et al.*, 1996), have been described and isolated from the surface sediments of an Antarctic meromictic lake and Russian Arctic tundra soil, respectively. *Msp. hansonii* requires saltwater for growth, whereas *Mbc. psychrophilus* does not need NaCl for growth. Several psychrotolerant Type Ia methanotrophs from various permanently cold habitats have been reported as new genera and species, including *Methylovulum psychrotolerans* (Oshkin *et al.*, 2016), *Methyloglobulus morosus *(Deutzmann *et al.*, 2014), *Methylovulum miyakonense* (Iguchi *et al.*, 2010), *Methyloprofundus sedimenti* WF1^T^ (Tavormina *et al.*, 2015), *Methylosoma difficile* (Rahalkar *et al.*, 2007), *Methylobacter tundripaludum* (Wartiainen *et al.*, 2006), and *Methylomonas scandinavica* (Kalyuzhnaya *et al.*, 1999). Three methanotrophic Type Ia bacteria, strain M200, *Methylomonas* strain M5 (Kip *et al.*, 2011), and *Methylomonas paludis* strain MG30 (Danilova *et al.*, 2013), were recently isolated from peat ecosystems. They were reported as being acid-tolerant (growth below a pH of 4.5). Furthermore, two sheathed and filamentous freshwater microorganisms, *Crenothrix polyspora* Cohn 1870 (Stoecker *et al.*, 2006) and *Clonothrix fusca* Roze 1896 (Vigliotta *et al.*, 2007), were identified and associated with gammaproteobacterial methane oxidizers. They appear to be psychrotolerant, but have not yet been grown in pure cultures.

Spitsbergen is the largest island of the high Arctic Svalbard archipelago, located north of Norway. It has a geologically active past and hot groundwater and glacial meltwater is mixed in several places, leading to karst springs at which the water stays above freezing temperature throughout the year (Lauritzen and Bottrell, 1994). One of the largest thermal springs on South Spitsbergen is Trollosen, located in the southernmost part (Fig. 1). Trollosen discharges water at a maximum rate of 18 m^3^ s^–1^, which varies with the seasons. Water temperature varies between 4 and 15°C. The smell of hydrogen sulphide (H_2_S) and the presence of organic slime in discharged water provides evidence of an active microbial community in the Trollosen aquifer (Lauritzen and Bottrell, 1994). In a previous study based on cultivation-independent analyses, sulphur-oxidizing chemolithotrophic *Proteobacteria* were found to dominate the bacterial community in this spring, with minor contributions by heterotrophs, including methane oxidizers (Reigstad *et al.*, 2011). In the present study, water and slime samples were collected to investigate methane-utilizing bacteria in the Trollosen spring. A novel cold-adapted methanotrophic bacterium belonging to the candidate family “*Methylomonadaceae*” of the class *Gammaproteobacteria* was recovered.

## Materials and Methods

### Sampling and growth conditions

In June 2006, approximately 40‍ ‍mL of water, containing white slimy filaments, was collected from the thermal spring, Trollosen (76°72'N, 16°28'E), in the South Spitsbergen National Park during a joint expedition with another group from the University of Bergen (Reigstad *et al.*, 2011). Samples were collected in Falcon tubes and stored at 8°C before being used as an inoculum for the enrichment and cultivation of methanotrophs. The *in situ* temperature and pH of the sample were 15°C and 6.8, respectively. The 3×1‍ ‍m cave opening of the spring is dominated by glacial meltwater from the nearby Vitkovskij glacier and discharged approximately 8 m^3^ s^–1^ at the time of sampling. On the day of sampling, the water contained (in decreasing order of concentrations in mM); Cl^–^, 56; Na, 2.76; Ca, 2.46; Mg, 0.81; ammonia, 0.19; SO_4_^2–^, 0.18; Li, 0.1; and lesser amounts of Al, Fe, B, Mn, As, Mo, and Ba. The amounts of nitrate and phosphate were below the detection limit (Reigstad *et al.*, 2011).

A previously described low-salt mineral medium (LMM) with pH 6.8 and vitamins was used for enrichment conditions (Islam *et al.*, 2015), except that KNO_3_ was replaced with NH_4_Cl (0.1‍ ‍g L^–1^). Thereafter, 2‍ ‍mL of the water sample was added directly to 20‍ ‍mL of LMM in a 120-mL serum bottle. The bottle was closed with butyl rubber caps and aluminium crimped seals. A mixture of methane (80%) (purity of methane, 99.5%; Yara Praxair), air (10%), and CO_2_ (10%) was added aseptically via a syringe into the headspace. The bottle was incubated at 18°C in the dark and without shaking for three weeks. The gas mixture was replaced every seven days. After the incubation, the enrichment culture became visibly turbid and was examined using a phase-contrast microscope (Eclipse E400 microscope; Nikon). Thereafter, 2‍ ‍mL of the culture was transferred to fresh LMM and re-incubated under the same conditions as those described above.

### Enrichment, isolation, and morphology

To recover a true methane oxidizer, the environmental enrichment sample was transferred five times into fresh LMM and incubated with methane, air, and CO_2_. Serial dilutions (10^–6^ to 10^–8^) were prepared and 0.1-mL aliquots were spread onto either gelrite (20‍ ‍g L^–1^, Gelzan^TM^ CM; Sigma-Aldrich) or agar (Difco) plates containing LMM. The plates were then incubated for four weeks at 18°C in jars filled with a methane/air (2:1) mixture. Individual colonies were collected, streaked onto fresh plates, and re-incubated for three weeks. A single colony was then selected and examined by phase-contrast microscopy. The selected colony was transferred to fresh liquid LMM and incubated for three weeks under the same growth conditions. After a pure culture was extracted, LMM was used for its routine cultivation at 15 and 18°C for two weeks. The purity of the culture was assessed by phase-contrast and electron microscopies, observations of single colony growth on plates, and a heterotrophic contamination test using Luria-Bertani broth (1 to 5% [v/v]) and glucose (10‍ ‍mM). In addition, growth was examined in the diluted and undiluted nitrate or ammonium mineral salt media used to cultivate the methanotrophs (Whittenbury *et al.*, 1970). Morphology was assessed using a phase-contrast microscope and Jeol-1230 electron microscope. Exponentially-grown cells were harvested by centrifugation and used to prepare ultrathin sections for transmission election microscopy (TEM), as described previously (Islam *et al.*, 2015).

### Utilizable carbon and nitrogen sources, pH, temperature, and salt concentration

Various organic compounds were tested as possible carbon and energy sources at a concentration of 10‍ ‍mM in fresh LMM. The following substrates were tested: glucose, acetate, pyruvate, lactate, malate, succinate, ethanol, sucrose, fructose, maltose, mannitol, sorbitol, Luria-Bertani broth, and yeast extract. Growth on methanol, methylamine, formate, and formaldehyde was tested at concentrations between 0.03% and 0.2% (v/v) in LMM. To prevent vaporization, bottles were capped with butyl-rubber stoppers. Growth was also tested in nitrogen-free LMM (without KNO_3_) and adjusted to pH 6.8 in triplicate, and the only nitrogen source was N_2_ from the air. After three weeks of incubation, resultant growth was evaluated. The growth temperature range was tested at 0, 2, 5, 10, 13, 15, 18, 20, 22, 25, and 30°C (at pH 6.8). The effects of pH on growth were investigated and the test for antibiotic sensitivity was performed as previously described (Islam *et al.*, 2020). The effects of salt concentrations on growth were also assessed by adding NaCl (0.1, 0.5, 1, 2.0, and 3% [w/v]) to LMM. Growth was assessed after two weeks of incubation using optimal culture conditions.

### Acetylene inhibition test, naphthalene assay, and fatty acid analysis

To assess the effects of acetylene, 4% of acetylene (v/v) was added into the headspace after five days of incubation with methane or methanol. Triplicate flasks were used for the methane oxidation inhibition test. One flask without acetylene was used as a control. The naphthalene-oxidation assay was performed as described previously (Islam *et al.*, 2020). A phospholipid fatty acid (PLFA) analysis of cells grown at 15°C was performed at the DSMZ (Deutsche Sammlung von Mikroorganismen und Zelkulturen GmbH).

### DNA isolation and 16S rRNA and functional gene analyses

Genomic DNA was isolated using the CTAB procedure (Murray and Thompson, 1980). The 16S rRNA gene was amplified as described previously (Islam *et al.*, 2008) using a Peltier thermal cycler (PTC-200; MJ Research). PCR products were purified and sequenced using the BigDye kit for automated DNA sequencers (ABI 3700 PE; Applied Biosystems). Amplification of the functional genes *pmoA*, *mmoX*, *mxaF*, *nifH* (nitrogenase iron protein), and *cbbL* (the large subunit of the ribulose-1,5-bisphosphate carboxylase/oxygenase, RubisCO) was performed using genomic DNA (Table S1). PCR was accomplished using Dynazyme™ High-Fidelity DNA Polymerase (Finnzymes) and the PCR program was previously described (Islam *et al.*, 2015). *Methylococcus capsulatus* (strain Bath) DNA was used as a positive control. MilliQ water was used as a negative control. The PCR products of the gene *pmoA* were cloned using pCR4-TOPO (Invitrogen) according to the manufacturer’s protocol. The four independent clones of the *pmoA* gene were sequenced and confirmed to be identical. The Southern blotting technique was utilized for the verification of pMMO and sMMO. DNA from *Mcc. capsulatus* (Bath) and *Methylacidiphilum kamchatkense* (Kam1) were used as positive and negative controls, respectively. Genomic DNA from the pure isolate was digested with EcoRI and HindIII. Fragments of DNA were separated using agarose gel electrophoresis and transferred onto Hybond-N nylon membranes (Amersham Biosciences). The probes utilized for hybridization were made by PCR using the *pmoA* and *mmoX* primer sets (Table S1) and labeled with [α-^32^P]dCTP using the DNA labeling kit (Amersham Biosciences) as previously described (Baxter *et al.*, 2002; Islam *et al.*, 2008).

### Phylogenetic analysis and accession numbers

The 16S rRNA sequences and deduced protein sequences of the functional genes (using the ExPASy Translate tool; https://web.expasy.org/translate) were evaluated with available sequences in the GenBank database using BLASTn and BLASTp (the NCBI tools), respectively (Table S2). 16S rRNA gene and deduced amino acid (PmoA) sequences were aligned using the CLUSTAL W algorithm. Phylogenetic trees were constructed using different methods, such as the Neighbor-Joining, Maximum Likelihood, and Minimum-Evolution methods, based on the following models: Kimura 2-parameter, Maximum composite likelihood, Tamura 3-parameter, Jukes-Cantor, Jones Taylor-Thornton (JTT), Dayhoff, and Poisson. These models were implemented in the MEGA7 software package (Kumar *et al.*, 2016). The sequences of the 16S rRNA, *pmoA*, *mxaF*, and *nifH* genes of strain TFB have been deposited in GenBank under accession numbers GQ130272, KX282487, KX282488, and KX282489, respectively.

## Results and Discussion

### Isolation of strain TFB and morphological characteristics

**Fig. 2.**
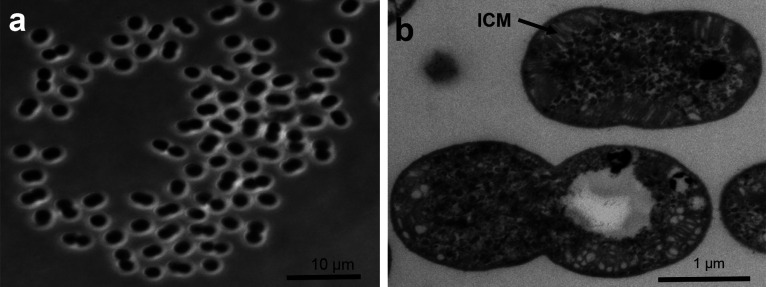
Cell morphology of strain TFB. (a) Phase-contrast micrograph of cells grown in LMM, containing methane, for 7 days. (b) Transmission electron micrograph showing intracytoplasmic membrane (ICM) systems. Bars, 10 μm (a) and 1 μm (b).

After three weeks of incubation at 18°C with methane, the enrichment was turbid, and phase-contrast microscopy revealed the presence of non-motile, straight, or curved rod-shaped cells, small cocci, and large coccoid cells with a thick mucous capsule. The large coccoid cells demonstrated poor growth with NH_4_Cl in enriched LMM. After five successive transfers into fresh LMM (with KNO_3_), the fraction of large coccoid cells was larger. After 3–4‍ ‍weeks of incubation, the following colonies were observed on gelrite plates: transparent (approximately 1.4 to 1.8‍ ‍mm in diameter), light yellow (approximately 0.5 to 1.0‍ ‍mm), small white (0.8 to 1‍ ‍mm), and large white (2.0 to 2.5‍ ‍mm). Transparent colonies were not present on the agar plates (even after five weeks of incubation). However, small and large white colonies were observed. To detect a true methane oxidizer, three different colonies were transferred to fresh LMM separately and incubated with methane, air, and CO_2_. Only a single transparent colony from the gelrite plate showed growth in methane after two weeks of incubation, whereas no growth was observed for the white or light-yellow colonies. A true cold-adapted methanotrophic isolate was obtained from the transparent colonies on the gelrite plates. The isolate, called TFB, was a large coccoid/elliptical rod with a length of 1.5–2.0 μm and diameter of 1.2–1.5 μm. The strain grew in LMM with methane or methanol (0.2%). It did not grow in the presence of methane or methanol under anaerobic conditions or in the absence of methane or methanol. These cells were non-motile and occurred individually or in pairs. An investigation by electron microscopy revealed the presence of intracellular membranes (ICM) stacked (as vesicular disks) throughout TFB cells, which is a common feature of the methanotroph family *Methylomonadaceae* (Fig. 2). Spores or cysts were not observed in TFB using TEM. The isolate had a polysaccharide-like matrix around it, which was also observed in the acid-tolerant methanotrophic strain M200 (Kip *et al.*, 2011). Similar bacteria, also described as large morphotype 2 psychrophilic methanotrophs, have been observed in enrichments from Russian Arctic tundra soil (Berestovskaya *et al.*, 2002). Moreover, these large cocci, with thick mucous capsules, grew at a pH and temperature between 5–7 and 5–10°C, respectively.

### Physiological characteristics

A comparison of the major characteristics of strain TFB with related cold-adapted (*e.g.* psychrophilic and psychrotolerant) and mesophilic Type Ia methanotrophs is shown in Table 1. Strain TFB utilized only methane and methanol as the sole carbon and energy sources, respectively. It did not grow on substrates containing multi-carbon compounds or in complex media, evincing obligate methanotrophy. Growth in methanol was observed at concentrations between 0.05 to 0.2% (v/v). Cells utilized nitrate and ammonium salts as nitrogen sources. In the absence of vitamins, TFB grew very poorly. The isolate grew on nitrogen-free LMM, demonstrating its ability to fix atmospheric N_2_, whereas growth was more optimal in LMM containing KNO_3_. Strain TFB grew between 2 and 22°C, with optimal growth occurring between 13 to 18°C (Fig. S1). This result implies that strain TFB is more psychrotolerant/psychrophilic than the acid-tolerant methanotrophic strain M200 and mesophilic *Mvm. miyakonense* HT12^T^. Although *Mvm. psychrotolerans* strains grew efficiently at temperatures lower than 10°C, their optimal growth temperature was 20–25°C (Oshkin *et al.*, 2016). No growth was observed at 25°C, indicating that strain TFB is not a member of the mesophilic Type Ia methanotrophs. The specific growth rate (μ) and doubling time at the optimum temperature were 0.017 h^–1^ and 18 h, respectively. At 15°C, strain TFB grew at a pH interval of 5.2–8.5; however, optimal growth was observed at a pH 6.8–7.2. It did not show any growth at pH 5.0 or 9.0, demonstrating that strain TFB is a neutrophilic bacterium rather than a member of the acid-tolerant or alkaline methanotrophs. The strain did not require excess NaCl for growth and grew favorably in LMM with 0.1% NaCl (w/v). When NaCl concentrations exceeded 0.5% (w/v), growth was inhibited. Acetylene acts as a suicide substrate for methanotrophs containing pMMO (Prior and Dalton, 1985). The effects of acetylene (4%) on TFB showed that growth and methane oxidation were completely suppressed, which confirmed the presence of pMMO as the functional methane oxidization enzyme. This result is consistent with previous findings obtained on other Type Ia, Type Ib, and verrucomicrobial methanotrophs (Bedard and Knowles, 1989; Islam *et al.*, 2008).

### Fatty acid profile

Strain TFB exhibited a unique fatty acid profile from the known psychrophilic and psychrotolerant methanotrophic genera of MOB (Table 2). The predominant fatty acids were C16:1*ω*5*c*, C14:0, and C16:1*ω*7*c*. The fatty acid C16:1*ω*5*c* is generally found in related psychrotolerant species, such as *Mvm. psychrotolerans* and the methanotrophic strain M200. The genera *Methyloterricola oryzae* 73a^T^ (a member of Type Ib in the family *Methylococcaceae*) contained 28.3% of C16:1*ω*5*c*, whereas strain TFB contained 49.65%. This was relatively high, and such a high percentage of C16:1*ω*5*c* has not previously been reported in any gammaproteobacterial methanotrophs described to date. A recent study reported that *Mvm. psychrotolerans* adapts to decreasing growth temperatures by increasing unsaturation in bulk fatty acids, including C14 and C16, as well as in hopanoids (Bale *et al.*, 2019). At 4°C, *Mvm. psychrotolerans* possesses 88–91% unsaturated fatty acids (mostly isomers of C16:1 and C14:1*ω*7), and 79–80% when grown at 20°C (Bale *et al.*, 2019). The high amount of C16:1*ω*5*c* in strain TFB indicates that it plays an important role in cold adaptation and may be used as a diagnostic feature in identifying cold-adapted methanotrophs and differentiating them from other mesophilic or thermotolerant methanotrophs. The amounts of C14:0, C16:1*ω*7*c*, and C16:0 were similar to those in other psychrophilic, psychrotolerant, and mesophilic methanotrophic genera. Additionally, C16:1*ω*7*c* in strain TFB showed a significant difference from *Mvm. myakonense* strain HT12^T^, while acid-tolerant methanotrophic strain M200 contained the fatty acid C16:1*ω*8*c* (37.4%), which was completely absent in strain TFB.

### Functional genes and phylogenetic analyses

Partial fragments of the functional genes *pmoA*, *mxaF*, and *nifH* were amplified and sequenced. The results obtained indicated a close relationship to corresponding genes in other Type Ia methanotrophs. PCR amplification trials on *mmoX* and subsequent Southern blotting (Table S3) yielded negative results that substantiated the negative naphthalene assay, thereby confirming the lack of genes encoding for sMMO in strain TFB. Furthermore, no feasible PCR band for the *cbbL* gene was detected, revealing that the strain may not utilize genes encoding RuBisCO for autotrophic CO_2_ fixation. The presence of RuBisCO in certain Type Ib methanotrophs (such as *Methylococcus*, *Methylocaldum*, *Methylomagnum*, *Methyloterricola*, *Methylospira*, and methanotrophic strains BFH1, BFH2, and LS7-MC) has been reported (Baxter *et al.*, 2002; Danilova *et al.*, 2013; Islam *et al.*, 2015; Islam *et al.*, 2016; Frindte *et al.*, 2017), whereas Type Ia methanotrophs, as well as some Type Ib methanotrophs (such as *Methylomonas*, *Methylomicrobium*, *Methylomarinum*, *Methylotetracoccus*, *Methyloparacoccus*, and *Methylogaea*), do not contain RuBisCO (Baxter *et al.*, 2002; Hirayama *et al.*, 2013; Ghashghavi *et al.*, 2019). The nearly complete 16S rRNA gene (1411 bases) and PmoA (169 amino acids) sequences of the methanotrophic strain TFB were used to construct phylogenetic trees.

**Fig. 3.**
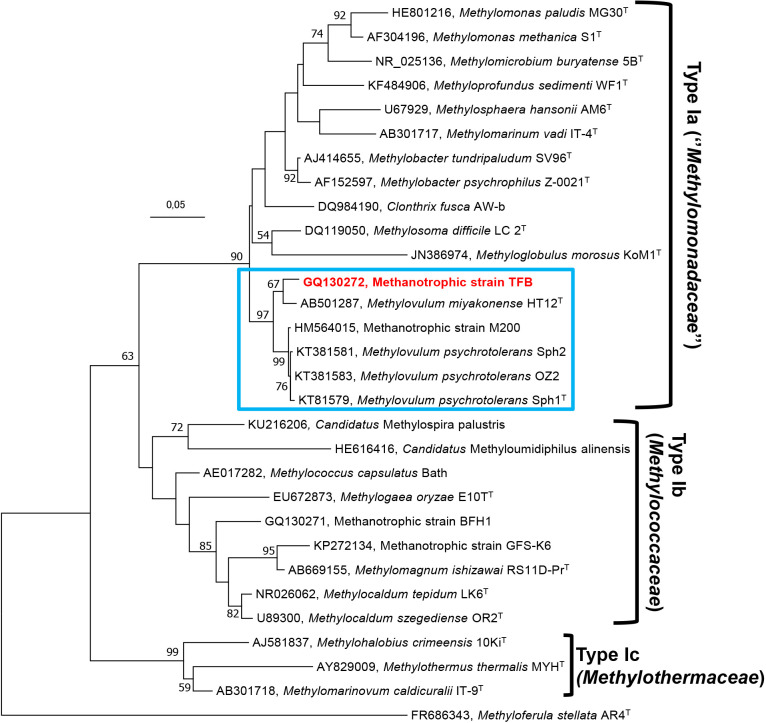
Molecular phylogenetic analysis (16S rRNA gene sequences) using the Maximum Likelihood method based on the Kimura 2-parameter model of strain TFB (indicated in bold red) and other related Type Ia, Type Ib, and Type Ic gammaproteobacterial methanotrophs. Evolutionary analyses were conducted in MEGA7. Nodes supported by bootstrap values (percentages of 1,000 data resamplings) ≥50% are shown at each node. The Type IIb methanotroph, *Methyloferula stellata* AR4 (FR686343) of the class *Alphaproteobacteria* (in the family *Beijerinkiaceae*) was used as an outgroup. Bar, 0.05 substitutions per nucleotide.

**Fig. 4.**
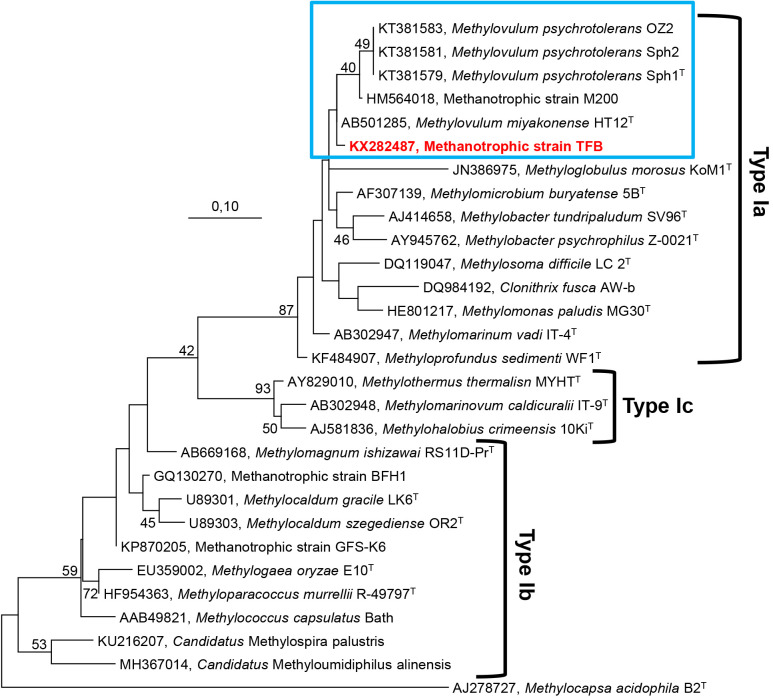
Molecular phylogenetic analysis (deduced amino acid sequences of the *pmoA* gene) of strain TFB (indicated in bold red) and other related Type Ia, Type Ib, and Type Ic gammaproteobacterial methanotrophs were inferred using the Maximum Likelihood method based on the Dayhoff matrix-based model. Initial tree(s) for the heuristic search were obtained automatically by applying the Maximum Parsimony method. Bootstrap values (1,000 replicates) less than 40% are not shown. Evolutionary analyses were conducted in MEGA7. The Type IIb methanotroph, *Methylocapsa acidiphila* B2^T^ (AJ278727) of the class *Alphaproteobacteria* (in the family *Beijerinkiaceae*) was used as outgroup. Bar, 0.10 substitutions per amino acid position.

Members of psychrotolerant Type Ia methanotrophic strains, such as *Msm. difficile* LC 2^T^ (Rahalkar *et al.*, 2007), *Mbc. tundripaludum* SV96^T^ (Wartiainen *et al.*, 2006), and *Mbc. psychrophilus* Z-0021^T^ (Omelchenko *et al.*, 1996), have 16S rRNA gene sequence identities of 93.0–93.6% to strain TFB. According to further 16S rRNA gene analyses, the methanotrophic bacterium M200 (Kip *et al.*, 2011), *Mvm. psychrotolerans* (strains Sph1^T^, Sph2, and OZ2), and *Mvm. miyakonense* HT12^T^ (Iguchi *et al.*, 2010) were identified as being closely related to strain TFB, with 96.6, 96.4–96.2, and 96.0% sequence similarities, respectively (Table S2). Furthermore, a phylogenetic analysis of the deduced amino acid sequence of *pmoA* showed high sequence similarities with strain M200 (98.8%), *Mvm. psychrotolerans* (98.8%), *Mvm. miyakonense* (100%), *Mbc. tundripaludum* (99.3%), *Mbc. psychrophilus* (97.5%), *Msm. difficile* (97.6%), and *C. fusca* (96.3%). The BLASTp search of MxaF protein sequences from the strain TFB showed the strongest associations with *Mvm. miyakonense* (96.6%), *Mvm. psychrotolerans* (96.6%), *Mbc. tundripaludum* (93.5%), and *Mgb. morosus* strain Kom1 (91.2%). NifH protein sequences showed associations with *Mvm. miyakonense*, *Mvm. psychrotolerans*, *Mbc. tundripaludum*, *Methylocucumis oryzae* (100%), and *Mpf. sedimenti* (95.2%). To establish the affiliation of two or more strains to the same genus, a minimum of 95% identity for 16S rRNA gene sequences is required (Schloss and Handelsman, 2004; Adekambi *et al.*, 2008). 16S rRNA gene sequence similarities (Table S2) between strain TFB (Arctic thermal spring), strain M200 (*Sphagnum* mosses), and *Mvm. miyakonense* HT12^T^ (forest soil) were 96.0–96.6%. This result suggests that strains TFB and M200 belong to the genus *Methylovulum*, or that they may denote two separate species, or even represent a new genus within Type Ia methanotrophs. Phylogenetic analyses of the 16S rRNA and *pmoA* genes of the strain TFB revealed that it, along with *Methylovulum* species (*Mvm. miyakonense* and *Mvm. psychrotolerans*) and the methanotrophic strain M200, most probably constitute a new cluster in the Type Ia methanotrophs of the class *Gammaproteobacteria* (Fig. 3, 4, S2, S3, S4, and S5). This phylogenetic assumption was facilitated using physiological characteristics (Table 1) and chemotaxonomic studies (Table 2). Furthermore, the 16S rRNA, *pmoA*, *mxaF*, and *nifH* sequences may contribute to the detection of related methane oxidizers from cold habitats and demonstrate how these cold-adapted bacteria are widely dispersed. Based on the 16S rRNA gene analysis, physiological properties, and fatty acid compositions, we propose that the colorless and neutrophilic methanotrophic strain TFB represents a new species of the genus *Methylovulum*. Strain TFB may be referred to as ‘*Methylovulum* sp. TFB’. However, the complete genus status of methanotrophic bacteria, namely *Mvm. miyakonense* HT12^T^, *Mvm. psychrotolerans* Sph1^T^, acid-tolerant methanotrophic strain M200, and the neutrophilic methanotrophic strain TFB require further investigations (*e.g.* comparisons including the whole genome) before decisions may be reached regarding the genus level.

## Citation

Islam, T., Larsen, Ø., and Birkeland, N.-K. (2020) A Novel Cold-adapted *Methylovulum* species, with a High C16:1ω5c Content, Isolated from an Arctic Thermal Spring in Spitsbergen. *Microbes Environ ***35**: ME20044.

https://doi.org/10.1264/jsme2.ME20044

## Supplementary Material

Supplementary Material

## Figures and Tables

**Fig. 1.**
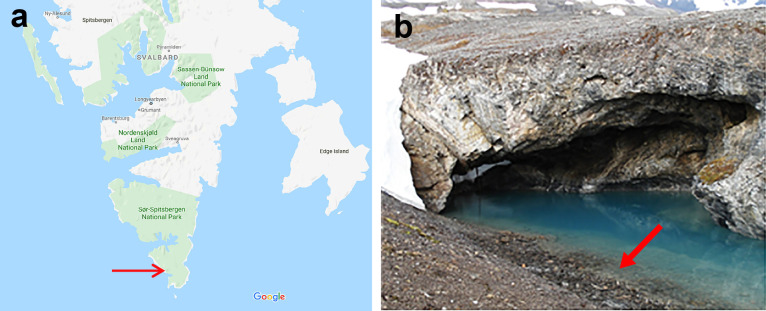
Sampling location. (a) Map of South Spitsbergen National Park in Svalbard designating the sampling site (https://www.google.com/maps). (b) A close-up photograph of the Arctic thermal spring (Trollosen). The arrow shows the sampling location.

**Table 1.** Comparisons of major characteristics of the cold-adapted strain TFB and Type Ia gammaproteobacterial methanotrophs. **Strains:** **1**) Strain TFB (this study); **2**) *Methylovulum psychrotolerans* (strains Sph1^T^, Sph2, and OZ2) (Oshkin *et al.*, 2016); **3**) Methanotrophic strain M200 (Kip *et al.*, 2011); **4**) *Methylovulum miyakonense* HT12^T^ (Iguchi *et al.*, 2010); **5**) *Methyloprofundus sedimenti* WF1^T^ (Tavormina *et al.*, 2015); **6**) *Methylosoma difficile* LC 2^T^ (Rahalkar *et al.*, 2007); **7**) *Methyloglobulus morosus* KoM1^T^ (Deutzmann *et al.*, 2014); **8**) *Methylobacter tundripaludum* SV96^T^ (Wartiainen *et al.*, 2006); and **9**) *Methylosphaera hansonii* ACAM 549^T^ (Bowman *et al.*, 1997). +, positive results; –, negative results; nr., not reported.

**Characteristics**	**1**	**2**	**3**	**4**	**5**	**6**	**7**	**8**	**9**
Cell morphology	Cocci, elliptical rods	Cocci	Cocci	Cocci or rods	Cocci, elliptical rods	Cocci, elliptical rods	Short rods	Rods	Cocci
pMMO	+	+	+	+	+	+	+	+	+
sMMO	–	–	–	+	–	–	–	–	–
*mxaF* gene	+	+	nr	+	+	+	+	+	nr
*nifH* gene/N_2_ fixation	+	+	+	+	+	+	+	+	+
Pigmentation	Transparent	Light pink	Pink	Pale brown	nr	Pale pink	Red pink	Pale pink	–^d^
**Growth temp. (opt.)**	**2–22 (13–18)**	**2–36 (20–25)**	**4–30** (nr)	**5–34 (24–32)**	**4–26 (18–23)**	**10–30 (25)**	**4–30 (20)**	**10–30 (23)**	**0–21 (10–13)**
pH range	5.2–8.5 (6.5–7.2)	4.0–8.9 (6.0–7.0)	4.1–7.0 (5.5)	6.0–7.5	6.0–8.0 (6.5–7.5)	5–9 (6–8)	5–8.5 (6–8)	5.5–7.9	7.0
Growth on methanol	+	+	+	+	+	+	+	–^c^	+
Vitamin required	+	–	–	–	+	+	–	–	–^e^
G+C content (mol%)^a^	nd	51.4–51.9	nr	49.3	40.5	49.9	47.7	47	43–46
G+C content (mol%)^b^	52.2	–	52.2	53.4	–	52.7	54	50.7	–
Source	Arctic thermal Spring	Cold methane seeps and freshwater lake	Acidic *Sphagnum* peat	Forest soil	Marine sediment	Lake sediment	Lake sediment	Wetland soil	Antarctic meromictic lake

^a^The G+C content was assessed by HPLC (DSMZ; Mesbah *et al.*, 1989; Kamagata and Mikami, 1991). ^b^16S rRNA, *pmoA*, *mxaF*, and *nifH* sequences were used to measure the DNA G+C content (mol%). ^c^Shows poor to no growth on methanol. ^d^Highly purified agar, agarose, and gelrite were likewise unsuccessful for pigmentation. ^e^Seawater required.

**Table 2.** Comparison of cellular fatty acid compositions of cold-adapted strain TFB with other psychrophilic, psychrotolerant, and mesophilic Type Ia and Type Ib methanotrophs. **1**) Strain TFB (this study); **2**) *Methylovulum psychrotolerans* (strains Sph1^T^, Sph2, and OZ2) (Oshkin *et al.*, 2016); **3**) Methanotrophic strain M200 (Kip *et al.*, 2011); **4**) *Methylovulum miyakonense* HT12^T^; **5**) *Methyloprofundus sedimenti* WF1^T^ (Tavormina *et al.*, 2015); **6**) *Methylosoma difficile* LC 2^T^ (Rahalkar *et al.*, 2007); **7**) *Methyloglobulus morosus* Kom1^T^ (Deutzmann *et al.*, 2014); **8**) *Methylobacter tundripaludum* SV96^T^ (Wartiainen *et al.*, 2006); **9**) *Methylosphaera hansonii* ACAM 549^T^ (Bowman *et al.*, 1997); **10**) *Methyloterricola oryzae* 73a^T^ (Frindte *et al.*, 2017); and **11**) *Methylococcaceae* strains BRS-k6, GFS-K6, and AK-K6 (Islam *et al.*, 2015). Values are given as a percentage of total fatty acids.

Fatty acids	**1**	**2**	**3**	**4**	**5**	**6**	**7**	**8**	**9**	**10**	**11**
C12:0	0.96	–	–	–	–	2.74	0.1	–	–	–	–
C13:1	1.53	–	–	–	–	–	–	–	–	–	–
**C14:0**	**18.28**	**7.1–9.3**	**7.5**	**34.2**	–	**8.55**	**0.7**	34.9	**2–3**	–	3.73–8.43
C15:0	1.22	–	0.7	2.97	–	0.79	–	**23.4**	1–2	–	–
C16:1*ω*8*c*	–	22.7–30.1	37.4	–	22.3	–	**55.3**	8.2	37–41	–	–
**C16:*****1ω7c***^a^	**16.52**	**22.5–33.0**	**14.9**	–	3–	**60.0**	**5.8**	–	**16–19**	**26.9**	57.93–69.41
C16:1*ω*6*c*	–	5.7–6.2	9.4	6.40	–	15.0	28.7	26.3	17–18	8.7	–
**C16:1*****ω*****5*****c***	**49.65**	**17.3–19.2**	**24.3**	–	–	–	–	–	–	**28.3**	**11.38–30.02**
C16:1*ω*7t	–	–	–	–	–	–	6.8	–	2–3	–	–
C16:1*ω*9t	–	–	–		26.9	–	–	–	–	–	–
C16:0	9.18	6.2–11.4	4.1	46.9	15	8.5	–	–	14–15	30.9	4.72–11.37
C16:1*ω*9*c*	–	–	–	–	28.8	–	–	–	–	–	–
C16:1*ω*10*c*	–	–	–	–	–	–	–	–	–	2,4	–
C16:1*ω*11*c*	–	–	–	–	–	2.44	–	–	–	–	–
C16:0 3OH	2.66	–	–	8.00	–	1.31	1.0	–	–	–	1.50–1.54
C16:2*ω*9,14	–	–	–		7.1	–	–	–	–	–	–
C18:1*ω*7*c*	–	0.7–1.2	–	–	–	–	0.9	–	1–2	1.7	–
C18:1*ω*9c	–	0–1.1	–	–	–	–	–	–	–	–	–
βOH-*n*C16:0	–	2.8–4.6	–	–	–	–	–	–	–	–	–
Growth temp. (°C)	15	20	nr*	24	23	25	20	20	10	30	25

* not reported
