# Supplementary material for: A Novel Cold-adapted Methylovulum species, with a High C16:1ω5c Content, Isolated from an Arctic Thermal Spring in Spitsbergen
Source: Microbes Environ. 2020 Jun 12;35(3):ME20044. doi: 10.1264/jsme2.ME20044 (PMC7511782; doi:10.1264/jsme2.ME20044)
Supplement: Supplementary file 1 — Supplementary Material [file 35_20044_s1.pdf]

Supplementary material

Table S1. Primers used for PCR amplification of functional genes.

Genes	Primer sequences (5'→ 3')	Product (bp)	Results	Annealing temp. (°C)	Ref.
pmoA	A189f: GGNGACTGGGACTTCTGG<br>Mb661: CCGGMGCAACGTCYTACC	510	+	55	1
mxaF	f1003: GCGGCACCAACTGGGGCTGGT<br>f1561: GGGCAGCATGAAGGGCTCCC	558	+	58	2
nifH	NifHf: GGHAARGGHGGHATHGGNAARTC<br>NifHr: GGCATNGCRAANCCVCCRCANAC	389	+	55	3
cbbL	McBCBBL 195F:<br>CTGCTGACCGACCTCGACTAMcBCBBL<br>706R: GTCACGTTGAGGTAGTGGCC	500	+	58	4
mmoX	mmoXf882:<br>GGCTCCAAGTTCAAGGTCGAGC<br>mmoXr1403:<br>TGGCACTCGTAGCGCTCCGGCTCG<br><br>*f92: GGCTGCAGAGCTTYAMCTGGA<br>*r1430: CGCCTCCCTCRTACTGYTCGAG	535<br><br><br><br><br>1335	−<br><br><br><br><br>−	55<br><br><br><br><br>55	5<br><br><br><br><br>6

*Probes used for Southern hybridization.

References:

1. Costello, A. M. and Lidström, M. E. (1999). Molecular Characterization of Functional and Phylogenetic Genes from Natural Populations of Methanotrophs in Lake Sediments. *Appl Environ Microbio*l **65**: 5066−5074.

2. McDonald, I. R. and Murrell, J. C. (1997). The methanol dehydrogenase structural gene mxaF and its use as a functional gene probe for methanotrophs and methylotrophs. *Appl Environ Microbiol* **63**: 3218−3224.

3. Mehta, M. P., Butterfield, D. A., and Baross, J. A., (2003). Phylogenetic Diversity of Nitrogenase (*nifH*) Genes in Deep-Sea and Hydrothermal Vent Environments of the Juan de Fuca Ridge. *Appl Environ Microbio*l **69**: 960−9704

4. Baster, N. J., Hirt, R. P., Bodrossy, L., Kovacs, K., L., Embley, T. M., Prosser, J. I., and Murrell, J. C. (2002). The ribulose-1,5-bisphosphate carboxylase/oxygenase gene cluster of *Methylococcus capsulatus* (Bath). *Arch Microbiol* **177**: 279−2895.

5. McDonald, I. R., Bodrossy, L., Chen, Y., and Murrell, J. C. (2008). Molecular ecology techniques for the study of aerobic methanotrophs. *Appl Environ Microbio*l **74**:1305−1315.

6. Islam, T., Jensen, S., Reigstad, L. J., Larsen, Ø. and Birkeland, N. K. (2008). Methane oxidation at 55°C and pH 2 by a thermoacidophilic bacterium belonging to the Verrucomicrobia phylum. *Proc Natl Acad Sci USA* **105**: 300−304.

Table S2. Pairwise sequence alignment analysis of the cold-adapted strain TFB (16S rRNA, *pmoA* and partially derived PmoA amino acid sequences) and other cultivated Type Ia MOB. Methods: Local alignment (Website: http://www.ebi.ac.uk/Tools/psa). Values are given as percentages. nr.; not reported.

**Strains**	**16S rRNA**	***pmoA***	**PmoA** (identity)	**PmoA** (similarity)
**Methanotrophic strain TFB** (this study)	100	100	100	100
Methanotrophic strain M200	96.6	91.1	95.5	98.8
Methylovulum psychrotolerans OZ2	96.4	89.4	95.3	98.8
Methylovulum psychrotolerans Sph2	96.2	89.4	95.3	98.8
Methylovulum psychrotolerans Sph1^T	96.0	89.4	95.3	98.8
Methylovulum miyakonense HT12^T	96.0	91.1	98.2	100
Clonothrix fusca strain AW-b	94.2	81.4	88.8	96.3
Methylobacter tundripaludum SV96^T	93.4	84.8	92.7	99.3
Methylobacter psychrophilus Z-0021^T	93.0	82.9	91.2	96.9
Acidophilic *Methylomonas* strain M5	92.1	80.1	88.0	94.6
Methylomicrobium album BG8^T	92.5	79.8	88.3	93.8
Methylomonas methanica S1^T	91.7	81.4	87.1	94.2
Methylosoma difficile LC 2^T	91.1	86.1	92.9	98.2
Methylosphaera hansonii ACAM 549^T	89.7	nr.	nr.	nr.
Methyloprofundus sedimenti WF1^T	89.7	82.2	89.9	97.0
Methylopracoccus murrelli R-49797^T	86.3	68.6	76.1	88.7

Table S3. Results of Southern hybridization for detection of radioactively labeled *pmoA* and *mmoX* probes.

**Strain**	***pmoA***	***mmoX***	**Ref.**
Strain TFB	+	−	This study
Methylococcus capsulatus strain Bath	+	+	1,2
Methylacidiphilum kamchatkense strain Kam1	−	−	3

References

1. Bodrossy, L., Kovács, K. L., McDonald, I. R., and Murrell, J. C. (1999). A novel thermophilic methane-oxidizing γ- Proteobacterium, *FEMS Microbiol Lett* **170**: 335-341.

2. Baxter, N. J., Hirt, R. P., Bodrossy, L., Kovacs, K. L., Embley, M. T., Prosser, J. I., and Murrell, J. C. (2002). The ribulose-1,5-bisphosphate carboxylase/oxygenase gene cluster of *Methylococcus capsulatus* (Bath). *Arch Microbiol* **177**: 279-289.

3. Islam, T., Jensen, S., Reigstad, L. J., Larsen, Ø. and Birkeland, N. K. (2008). Methane oxidation at 55 ^{o}C and pH 2 by a thermoacidophilic bacterium belonging to the Verrucomicrobia phylum. *Proc Natl Acad Sci USA* **105**: 300−304.

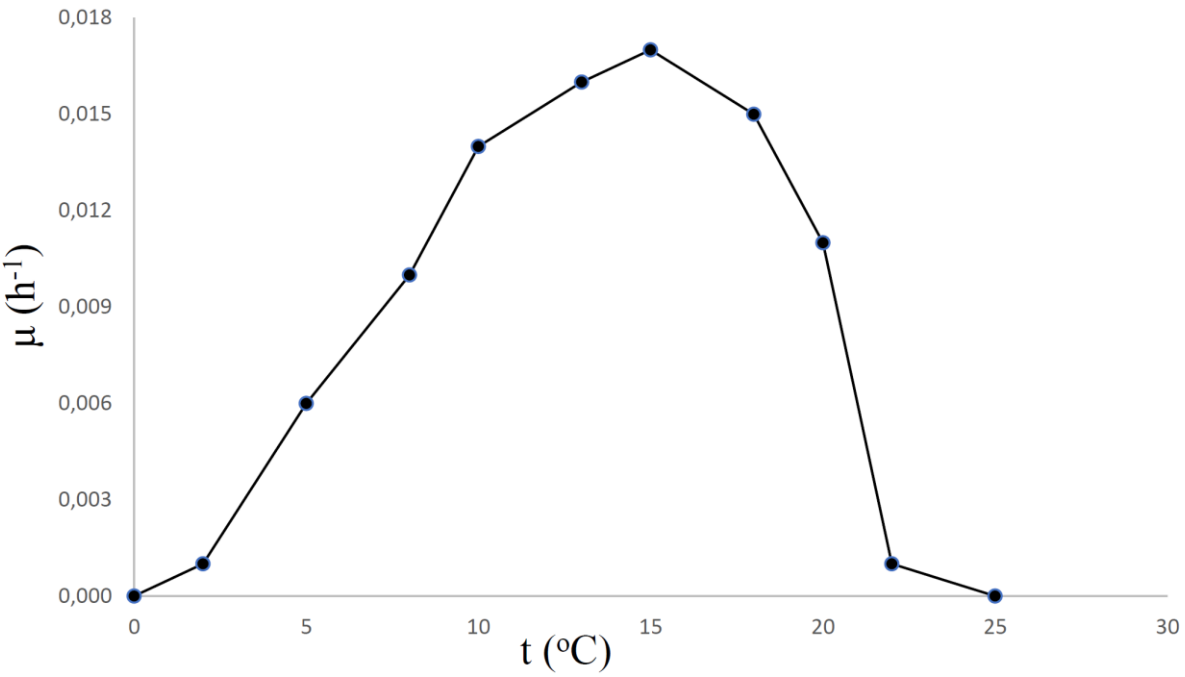

Fig. S1. Relation of temperature to specific growth rate (μ) of strain TFB.

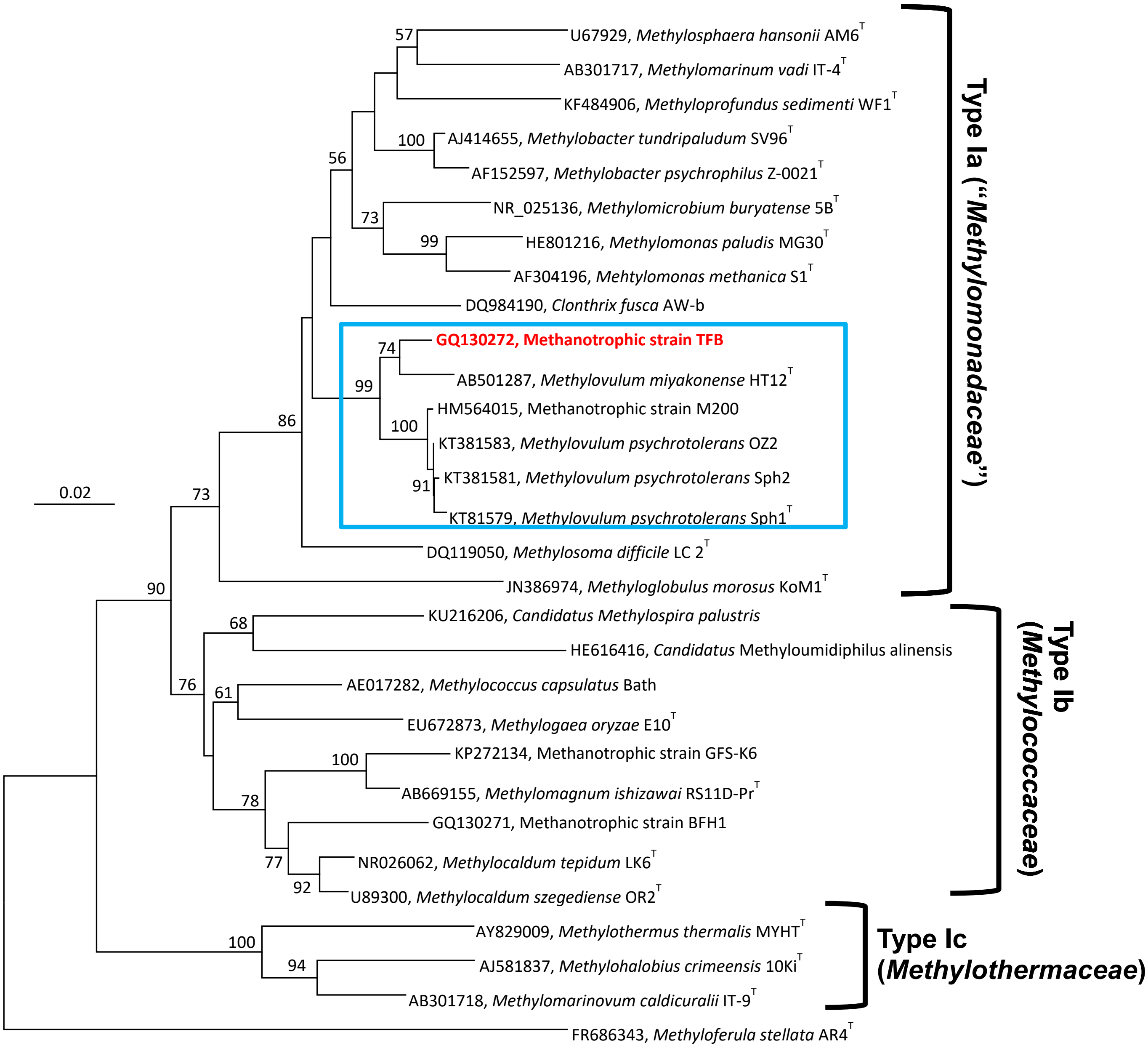

Fig. S2. 16S rRNA gene-based phylogenetic tree (the neighbor-joining algorithm) of strain TFB (indicated in bold red) and representatives from the Type Ia, Type Ib, and Type Ic methanotrophic strains of the class *Gammaproteobacteria* using the MEGA7 software package. Bootstrap values (percentages of 1000 data resamplings) ≥50% are shown at each node. The evolutionary distances were computed using the Maximum Composite Likelihood method and are in the units of the number of base substitutions per site. The Type IIb methanotroph, *Methyloferula stellata* AR4 (FR686343) of the class *Alphaproteobacteria* (in the family *Beijerinkiaceae*), was used as an outgroup.

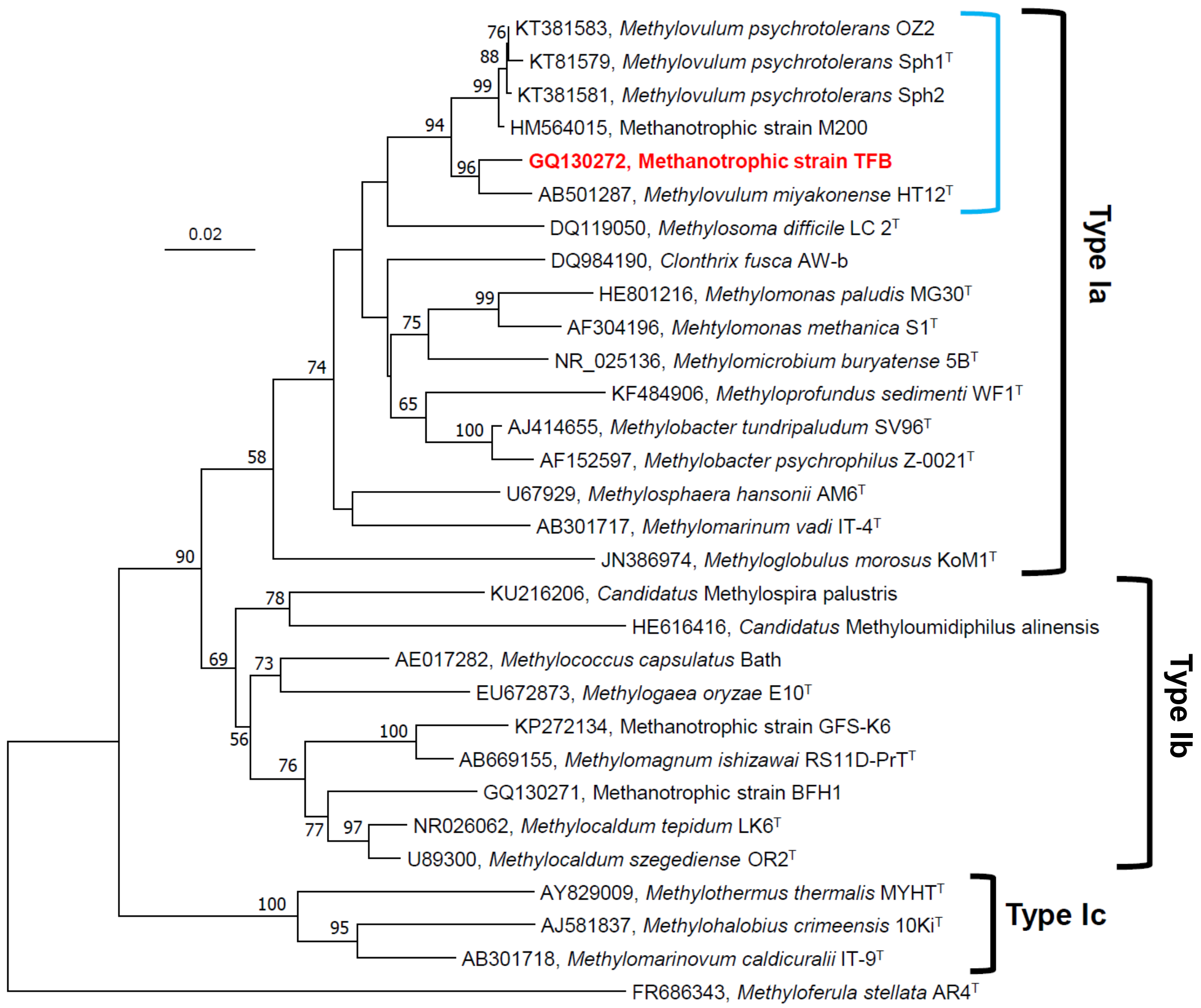

Fig. S4. Molecular Phylogenetic analysis (16S rRNA gene sequences) of strain TFB (indicated in bold red) and representatives from the Type Ia, Type Ib, and Type Ic methanotrophic strains of the class *Gammaproteobacteria* was inferred using the Minimum Evolution method. The evolutionary distances were computed using the Jukes-Cantor method. The Neighbor-joining algorithm was used to generate the initial tree. Evolutionary analyses were conducted in MEGA7. Bootstrap values (percentages of 1000 data resamplings) ≥50% are shown at each node. The Type IIb methanotroph, *Methyloferula stellata* AR4 (FR686343) of the class *Alphaproteobacteria* (in the family *Beijerinkiaceae*), was used as outgroup.

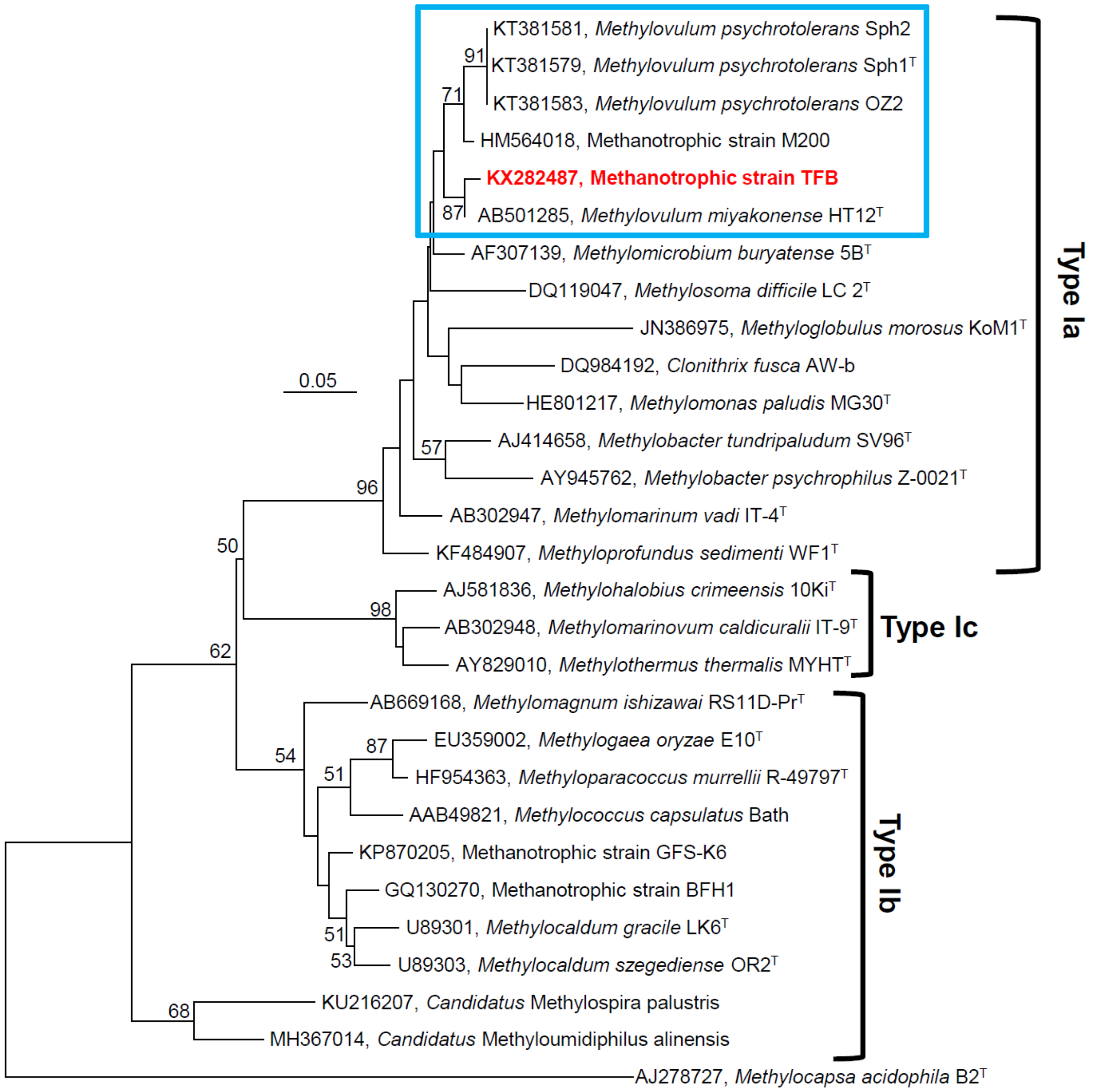

Fig. S4. Phylogenetic tree of deduced amino acid sequences (PmoA) of strain TFB (indicated in bold red) and representatives from the Type Ia, Type Ib, and Type Ic methanotrophic strains of the class *Gammaproteobacteria* using the MEGA7 software package. The evolutionary history was inferred using the Neighbor-Joining method. The evolutionary distances were computed using the Poisson correction method. Bootstrap values (percentages of 1000 data resamplings) ≥50% are shown at each node. The Type IIb methanotroph, *Methylocapsa acidiphila* B2^{T} (AJ278727) of the class *Alphaproteobacteria* (in the family *Beijerinkiaceae*), was used as an outgroup.

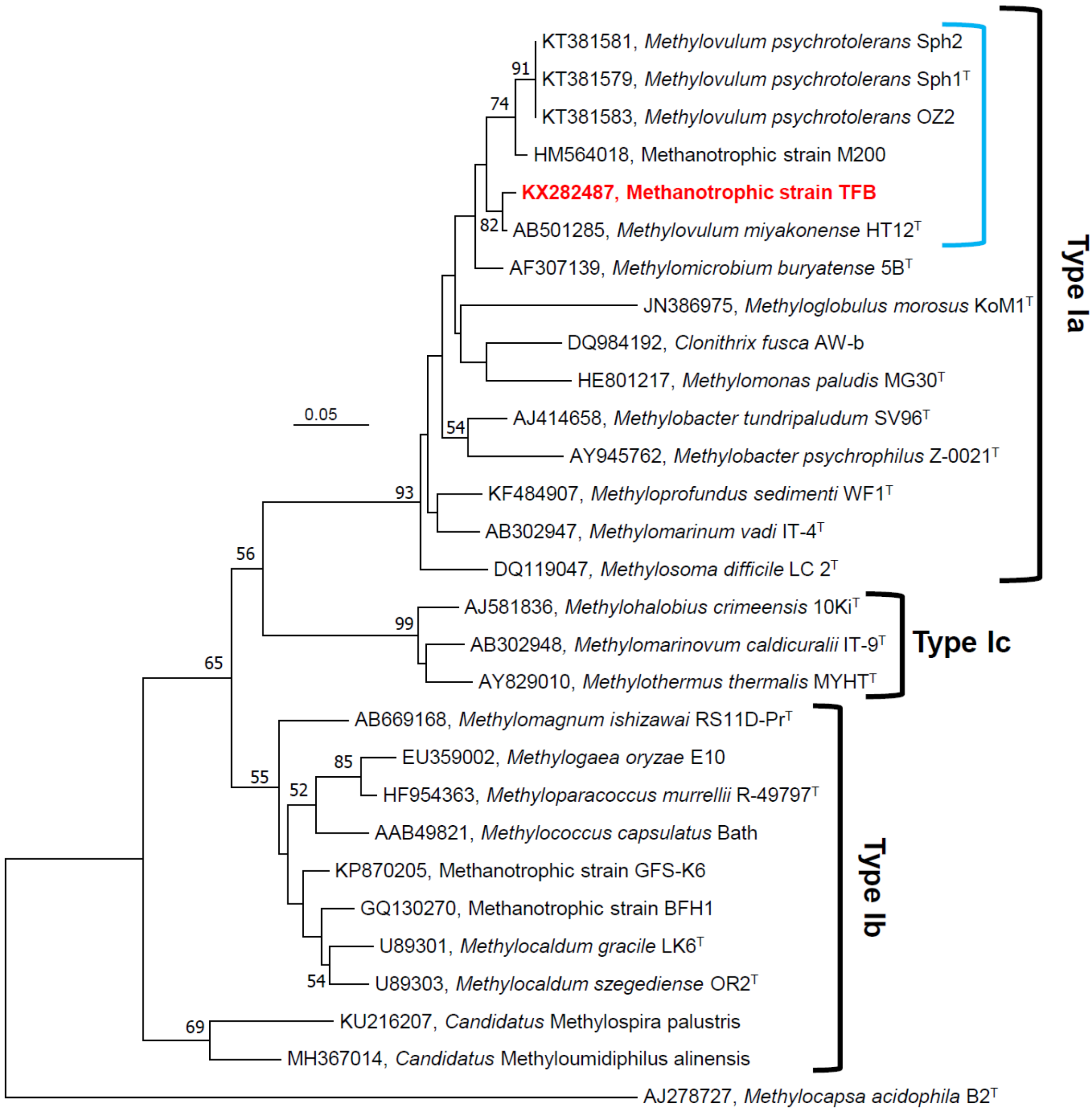

Fig. S5. Molecular phylogenetic analysis (deduced amino acid sequences of the *pmoA* gene) of strain TFB (indicated bold red) and other related gammaproteobacterial methanotrophs was inferred using the Minimum Evolution method. The evolutionary distances were computed using the JTT matrix-based method. Evolutionary analyses were conducted in MEGA7. Bootstrap values (percentages of 1000 data resamplings) ≥50% are shown at each node. The Type IIb methanotroph, *Methylocapsa acidiphila* B2^{T} (AJ278727) of the class *Alphaproteobacteria* (in the family *Beijerinkiaceae*), was used as outgroup.
